# Astragalus mongholicus bunge and panax notoginseng formula (A&P) improves renal fibrosis in UUO mice *via* inhibiting the long non-coding RNA A330074K22Rik and downregulating ferroptosis signaling

**DOI:** 10.1186/s12906-024-04557-4

**Published:** 2024-07-19

**Authors:** Xia Zhong, Yue Huang, Jian Jia, Jian Liu, Hongwei Su, Qiongdan Hu, Ruizhi Tan, Li Wang

**Affiliations:** 1 Research Center of Integrated Traditional Chinese and Western Medicine, The Affiliated Traditional Chinese Medicine Hospital, Southwest Medical University, 187 Chunhui Avenue, Longma-Tan District, Luzhou, Sichuan China; 2 Department of Nephrology, The Affiliated Traditional Medicine Hospital, Southwest Medical University, Luzhou, China; 3 Department of Urology, The Affiliated Traditional Medicine Hospital, Southwest Medical University, Luzhou, China; 4 Southwest Medical University, Luzhou, China

**Keywords:** Renal fibrosis, Chinese medicine, Ferroptosis, LncRNA, Astragalus mongholicus Bunge and Panax notoginseng formula (A&P)

## Abstract

**Background:**

Chronic kidney disease (CKD) and its associated end-stage renal disease (ESRD) are significant health problems that pose a threat to human well-being. Renal fibrosis is a common feature and ultimate pathological outcome of various CKD leading to ESRD. The Astragalus mongholicus Bunge and Panax notoginseng formula (A&P) is a refined compound formulated by our research group, which has been clinically administered for over a decade and has demonstrated the ability to improve the inflammatory state of various acute or chronic kidney diseases. However, the underlying mechanism by which A&P ameliorates renal fibrosis remains unclear.

**Methods:**

We established a mouse model by surgically ligating the unilateral ureter to induce renal injury in vivo. And we utilized renal in situ electroporation of a plasmid with low LncRNA A33 expression to establish the unilateral ureteral obstruction(UUO)mouse model. In vitro, we stimulated primary tubular epithelial cells(pTEC) injury using TGF-β1, siRNA-A33, and pcDNA3.1-A33 plasmids were transfected into pTECs to respectively knockdown and overexpress LncRNA A33, and both in vitro and in vivo models were intervened with A&P.

**Results:**

The results demonstrated that A&P effectively alleviated renal fibrosis in mice. Subsequent findings indicated high expression of LncRNA A33 in the kidneys of UUO mice and TGF-β1-induced renal tubular cells. In situ, renal electroporation of a plasmid with reduced LncRNA A33 expression revealed that inhibiting LncRNA A33 significantly improved renal fibrosis in UUO mice. Moreover, A&P effectively suppressed LncRNA A33 expression both in vitro and in vivo. Subsequent downregulation of LncRNA A33 in renal tubular epithelial cells resulted in the downregulation of numerous fibrotic markers, a significant inhibition of LncRNA A33, and a notable reduction in downstream ferroptosis signaling. Cell experiments demonstrated that A&P improved renal fibrosis in UUO mice by inhibiting LncRNA A33 and downregulating ferroptosis signaling.

**Conclusion:**

Through the inhibition of LncRNA A33 and subsequent downregulation of ferroptosis signaling, A&P showed potential as a therapeutic approach for improving renal fibrosis in UUO mice, providing a potential treatment avenue for CKD.

**Supplementary Information:**

The online version contains supplementary material available at 10.1186/s12906-024-04557-4.

## Introduction

CKD and ESRD are significant health conditions that pose a threat to human well-being. Renal fibrosis is a common characteristic and the final pathological manifestation in various chronic kidney diseases leading to end-stage renal disease. The progression of this pathological process is influenced by multiple factors, including inflammation, oxidative stress, cell apoptosis, proliferation of fibroblast cells, and epithelial-to-mesenchymal transition [1]. Due to the intricate mechanisms underlying CKD, specific drugs targeting the condition have yet to be developed. The results of a 2012 epidemiological survey in China revealed a CKD prevalence rate of 10.8% among adults aged 18 and above. Based on this estimation, China is projected to have over 120 million CKD patients, of whom approximately 2% will progress to ESRD. This alarming situation necessitates urgent attention [2]. Therefore, it is significant to elucidate the pathogenic mechanisms of CKD and explore effective treatment methods.

**Fig. 1:**
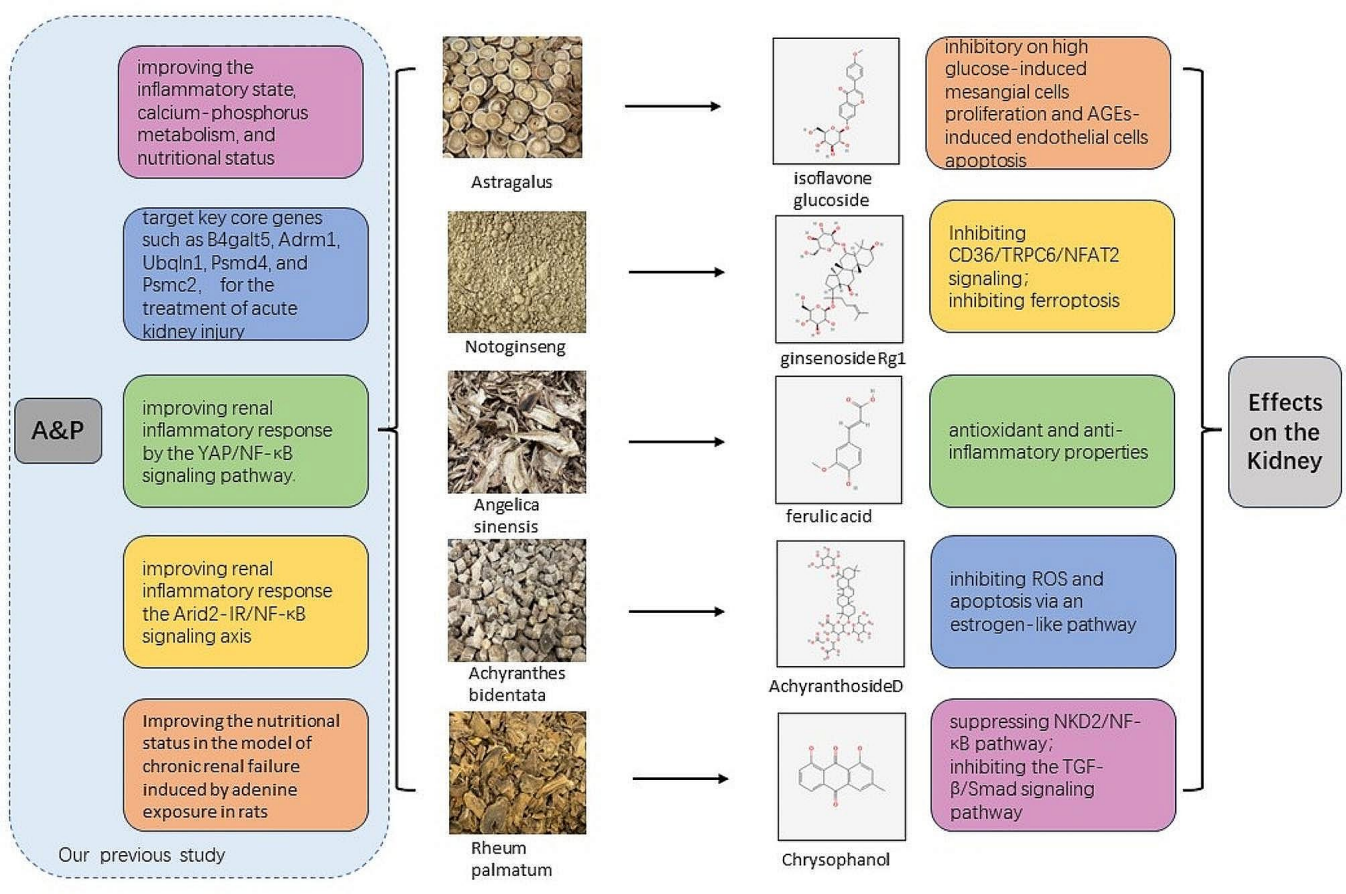
Our previous study about A&P and the impact of key TCM components on renal function

A&P is a refined traditional Chinese medicine formulation developed by our research team, which has been used in clinical practice for over a decade. It has demonstrated proven therapeutic efficacy, a high safety profile, and notable advantages [3]. Previous studies have reported that A&P can improve the inflammatory state, calcium-phosphorus metabolism, and nutritional status in hemodialysis patients, thereby protecting residual kidney function [4]. Animal experiments have shown that A&P may target key core genes such as B4galt5, Adrm1, Ubqln1, Psmd4, and Psmc2, which are involved in glycosylation modification or proteasome-related metabolic pathways for the treatment of acute kidney injury induced by ischemia-reperfusion injury [5]. In our previous in vitro work, A&P improves renal mesangial cell damage in diabetic nephropathy by inhibiting the inflammatory response of infiltrated macrophages [6]. And it combined With bifidobacterium contribute a renoprotective effect in chronic kidney disease through inhibiting macrophage inflammatory response in kidney and intestine [7]. Moreover, A&P also can significantly improve renal inflammatory response in mice with acute kidney injury induced by cisplatin, and this molecular mechanism may be associated with the regulation of LncRNA9884 expression, thereby affecting the YAP/NF-κB signaling pathway [8]. In diabetic nephropathy mice, A&P has been found to improve renal inflammatory response, possibly through the regulation of the Arid2-IR/NF-κB signaling axis [9]. Additionally, A&P has been shown to significantly improve the nutritional status in the model of chronic renal failure induced by adenine exposure in rats [10]. The first figure presents the research outcomes related to A&P and shows the impact of key traditional Chinese medicine components on renal function (Fig. 1). In summary, A&P and its main components have demonstrated definite clinical efficacy. However, the specific effects and mechanisms of A&P on CKD remain unclear. Therefore, we aim to investigate its role in both in vitro cellular systems and in vivo animal models.

LncRNA is a type of RNA sequence with a length greater than 200 nucleotides that do not encode proteins. Initially considered as a byproduct of RNA polymerase II transcription, lacking biological functionality, it was in 1991 that a paper published in Nature and other journals confirmed the involvement of Xist in the regulation of X chromosome inactivation. Subsequent research has increasingly demonstrated the pivotal role of Long Non-Coding RNAs (LncRNA) in numerous biological processes. The development of next-generation sequencing technologies has facilitated the discovery of a plethora of LncRNAs [11, 12]. The known functions of lncRNAs include direct interactions with RNA polymerase II, pre-initiation complexes, transcription factors, and regulatory factors to interfere with transcription processes [13]. Over the past decade, research has shown a close relationship between lncRNAs and renal fibrosis. For example, LncRNA NEAT1 is involved in regulating the ERK1/2 signaling pathway to protect the kidneys from the effects of epithelial-mesenchymal transition (EMT) and renal fibrosis [14]. LncRNA GAS5 can mediate the reduction of renal damage and fibrosis in diabetic rats through MMP9 ^15^. LncRNA1700020I14Rik can attenuate the proliferation and fibrosis of diabetic kidney cells through the miR-34a-5p/Sirt1/HIF-1α signaling pathway [15]. In early studies, RNA sequencing revealed high expression of LncRNA A33 in the kidney tissue of UUO mice [16]. However, there is currently hardly any literature reporting the specific role and mechanism of LncRNA A33.

Ferroptosis, a regulated form of cell death, has been implicated in pathogenic cell death associated with mammalian degenerative diseases, cancer, stroke, cerebral hemorrhage, traumatic brain injury, ischemia-reperfusion injury, and renal degeneration [17]. Moreover, an increasing body of research suggests that non-coding RNAs, particularly miRNAs, lncRNAs, and circRNAs, can interfere with the progression of ferroptosis by directly or indirectly regulating ferroptosis-related genes or proteins [18].

This study seeks to elucidate the crucial role of LncRNA A33 in renal fibrosis in both UUO mice and TGF-β1-induced pTEC. Furthermore, our primary emphasis is on examining how A&R intervenes to ameliorate renal fibrosis in UUO mice by inhibiting LncRNA A33 and downregulating ferroptosis signaling. The overarching objective is to identify potential therapeutic agents for the treatment of CKD and to offer novel insights into treatment strategies.

## Materials and methods

### Drug preparation and administration

A&P concentrate granules (30 g of Astragalus propinquus Schischkin, 10 g of Panax notoginseng, 30 g of Angelica sinensis, 30 g of Achyranthes bidentata, and 6 g of Rheum palmatum) were obtained from the Preparation Department of the Affiliated Traditional Chinese Medicine Hospital at Southwest Medical University. The drugs were dissolved in physiological saline and administered via oral gavage twice daily at a dose of 4.5 mg/kg per administration (calculated based on the conversion formula from experimental animals to human dosage). The control group received an equivalent volume of physiological saline at the same frequency via oral gavage. Different concentrations of A&P (5%, 10%, 20%, 25%, and 30% ) enriched serum were prepared using rats to facilitate in vitro cell experiments intervention.

### Extraction of pTECs and establishment of Fibrosis Model in vitro

For this study, C57BL/6 male mice below 23 days of age were selected. The cortex and medulla of the kidneys were separated, and the cortex was finely minced to extract cells. The cell suspension was repeatedly pipetted through a pipette tip several times. A new culture dish with a 70 μm mesh sieve was used to filter the cells. The 70 μm sieve and kidney tissue fragments were repeatedly washed with a stopping solution to terminate digestion, followed by centrifugation of the filtrate. The supernatant was removed, and the cell pellet in the centrifuge tube was resuspended in a preheated basic culture medium. After 1–2 min of settling, the supernatant was discarded, and a fresh primary culture medium pre-equilibrated at 37 °C was added. The cells were placed in a 37 °C, 5% CO2 incubator, and the medium was changed after 72 h. Treatment was initiated when cell confluence reached approximately 80%. Subsequently, cells were harvested for flow cytometry analysis. Cells with Anti-Cytokeratin 18 > 90% and Anti-Podocin < 5% were considered qualified for experiments. The cell model group received an addition of 5 ng/ml TGF-β1 for model construction.

### Modulation of LncRNA A33 expression in pTECs

The downregulation and overexpression of LncRNA A33 in primary tubular epithelial cells (pTECs) were achieved using Lipofectamine RNAiMAX reagent (Invitrogen, USA). siRNA-A33 was transfected into pTECs using the following sequence: Forward, 5’-GCGUAGCUUGCUCAUAUT-3’; Reverse, 5′-AUAUAGAGCAGAGUACCGCTT (Synthesized by Sangon Biotech, China). Additionally, the pcDNA3.1-A33 plasmid (constructed by FulenGen Corporation) was transfected into pTECs using Lipofectamine 3000 reagent to induce A33 overexpression.

### Animal model establishment and grouping

A total of 30 male c57BL/6 mice at the age of 6 weeks were purchased from Chongqing Tengxin biology Co.,ltd. After 12 h of fasting and water deprivation, the mice were anesthetized by intraperitoneal injection of sodium pentobarbital (50 mg/kg). The mice were subjected to ureteral ligation near the renal hilum, followed by a second ligation away from the renal hilum. The mice were randomly divided into the following groups (*n* = 10): Ctrl group (normal control), UUO group (model control), UUO with lncRNA A33 knockdown group (UUOsh), UUO with empty vector group (UUOep), and UUO with A&P group (UUO + L or H). Gene organ-targeting delivery of lncRNA A33 knockdown.

Gene organ-targeting delivery of lncRNA A33 knockdown: A 32G needle was used to inject 30 µg of lncRNA A33 shRNA knockdown plasmid into the renal pelvis. In the control group, an empty vector was injected. Subsequently, the kidney was clamped using a specialized electroporation instrument. The kidney was subjected to 100 V voltage, 50 ms pulses, for a total of 6 times. After 7 days, the mice were anesthetized with isofurane and sacrifcedand, the ligated kidneys were collected for further analysis. All animal experiments were conducted according to the guidelines of the Ethics Committee of Southwest Medical University (No 20211126-033). Animal care and procedures comply with the ethical guidelines issued by the International Scientifc Committee on Experimental Animals (ICLAS).

### Pathological examination

HE staining: Deparaffinization was performed using xylene, followed by a series of ethanol washes until rehydration was achieved. The sections were stained with hematoxylin and eosin (HE), rinsed with tap water, differentiated with hydrochloric acid-ethanol solution, and counterstained with eosin for 2 min. After a final rinse with running water, the samples were dehydrated in ethanol and cleared with xylene before being mounted with a neutral mounting medium.

Masson’s staining: Deparaffinization was performed using xylene, followed by a series of ethanol washes until rehydration was achieved. The sections were then incubated with potassium dichromate overnight, rinsed with running water, stained with Weigert’s iron hematoxylin, differentiated with 1% hydrochloric acid alcohol, and reblued. They were then stained with ponceau acid fuchsin, differentiated with phosphomolybdic acid, rinsed with 1% acetic acid, dehydrated in ethanol, and cleared with xylene before being mounted with neutral resin. All sections were examined under the Leica DM4B upright digital research microscope (Leica, DMC6200, Germany).

### Immunohistochemistry

Paraffin sections of kidney tissue were deparaffinized and rehydrated with water. Antigen retrieval was performed using a sodium citrate buffer, followed by cooling to room temperature and PBS washes. After treatment with hydrogen peroxide solution at room temperature and a subsequent PBS wash, the sections were blocked for 1 h. Primary antibodies (α-SMA, Fn, Collagen1, KIM1, etc.) were then added and incubated overnight at 4 °C. Counterstaining was performed with hematoxylin. Brown particles were observed under an optical microscope. A negative control was prepared by omitting the primary antibody treatment, and the positivity rate (PR) was calculated as the percentage of positive area or cells relative to the total area or cell count.

### Real-time PCR

Reverse transcription reactions were performed using the cDNA Synthesis Kit (Vazyme) according to the manufacturer’s instructions. Expression levels of various RNA molecules (α-SMA, Fn, lncRNA A330074K22Rik, GPX4, COX2, etc.) in each group were determined using the protocols provided in the fluorescent real-time quantitative PCR reagent kit instructions.

### Protein extraction and western blotting

Total protein from kidney tissue was extracted using a conventional method. The protein concentration was determined using the Bio-Rad DC protein assay reagent. An appropriate volume of protein samples was loaded onto an SDS-PAGE gel, followed by electrophoresis. Subsequently, the proteins were transferred to a membrane, blocked, and incubated with primary antibodies (α-SMA, Fn, KIM1, GPX4, COX2, etc.) overnight at 4 °C. After washing with TBST, the membrane was incubated with appropriate secondary antibodies. The protein bands were then visualized using an ECL detection system.Statistical analysis was performed with GraphPad Prism 9.0 software(GraphPad Software Inc CA, USA). The *p* value < 0.05 was statistically significant.

## Results

### A&P significantly improves renal fibrosis in UUO mice

**Fig. 2:**
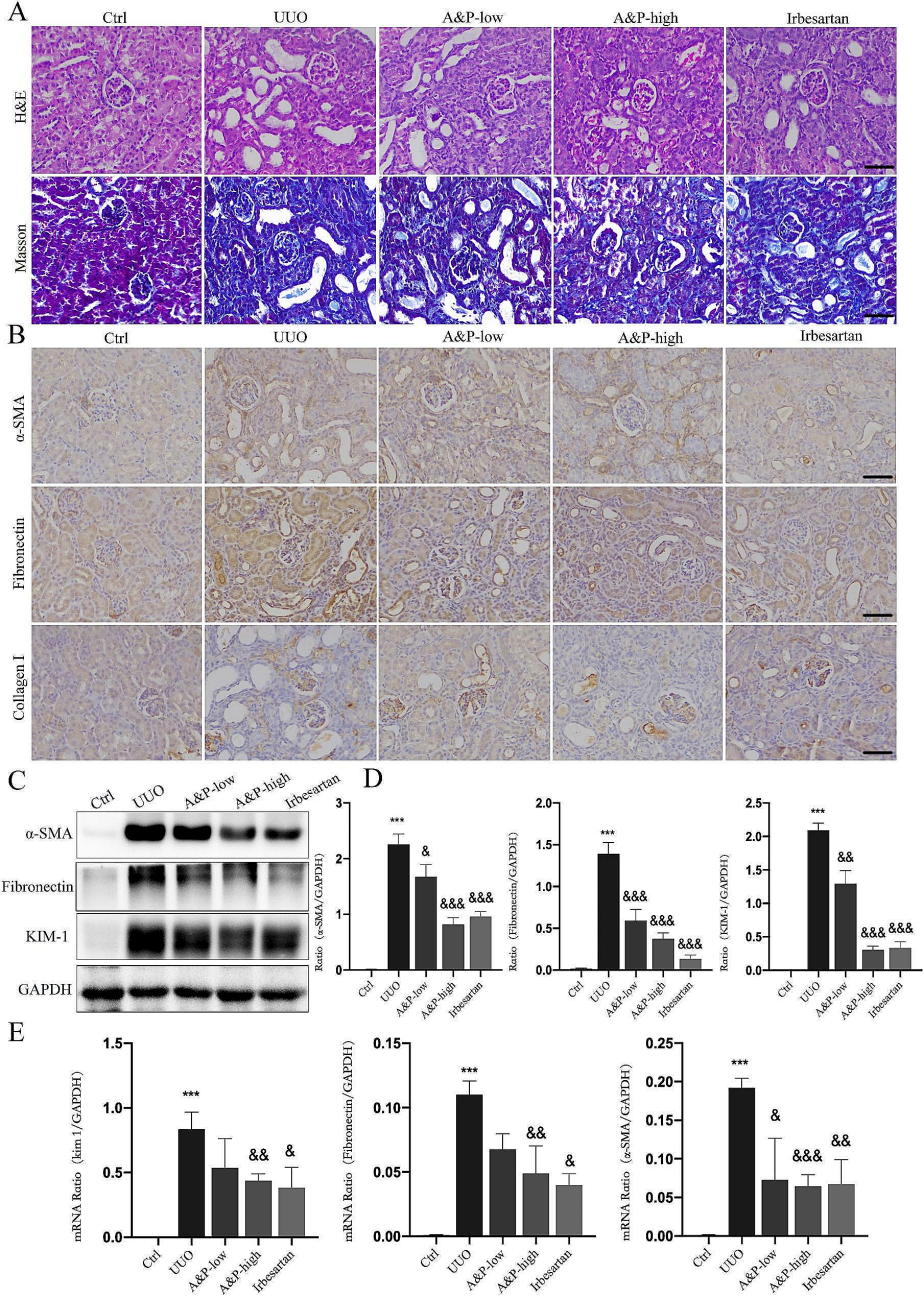
A&P Significantly Improves Renal Fibrosis in UUO Mice. (**A**) Results of H&E and Masson staining in each group indicate a significant improvement in renal structural damage with A&P in UUO mice. (**B**) Immunohistochemistry results demonstrate that A&P reduces renal injury and collagen deposition (α-SMA, Fibronectin, Collagen1) in UUO mice. (**C**, **D**, **E**) Real-time PCR and Western blot analysis reveal the expression levels of fibrosis-related proteins and mRNA (α-SMA, Fibronectin, KIM-1) in the kidneys. Compared with Ctrl group, ***P < 0.001. Compared with UUO group, &*P* < 0.05, &&<0.01, &&&*P* < 0.001

To investigate the protective and anti-fibrotic effects of A&P on the kidneys, we successfully established a mouse model of chronic fibrosis by UUO. Kidney tissue structure and pathological changes were examined using HE and Masson’s staining. The control group (Ctrl) showed normal structure of glomeruli and renal tubules. In the UUO group, severe renal injury was observed, characterized by increased glomerular volume, partial detachment interstitial fibrosis, and inflammatory cell infiltration. Additionally, some renal tubules exhibited dilation. However, both low and high doses of A&P treatment significantly inhibited renal structural damage, as observed in the renal pathology (Fig. 2A). Additionally, immunohistochemistry was performed to assess the expression of α-SMA, Fibronectin, and Collagen1 in renal tissues. The UUO mice showed increased deposition of these fibrotic markers, while treatment with losartan and A&P (low dose) reduced the deposition, and A&P (high dose) treatment resulted in a significant reduction (Fig. 2B). Furthermore, real-time PCR and protein immunoblotting analysis demonstrated that treatment with both high and low doses of A&P led to a downregulation of activated α-SMA, Fibronectin, and KIM-1 fibrotic factors protein levels and mRNA expression (Fig. 2C-E). The results indicated that UUO group mice exhibited tubular injury, accompanied by extensive collagen deposition, while the expression of fibrosis markers was significantly reversed in the high-dose A&P group, which was superior to the Irbesartan group.

### Inhibition of A33 in kidney of UUO ameliorated renal injury and fibrosis

**Fig. 3:**
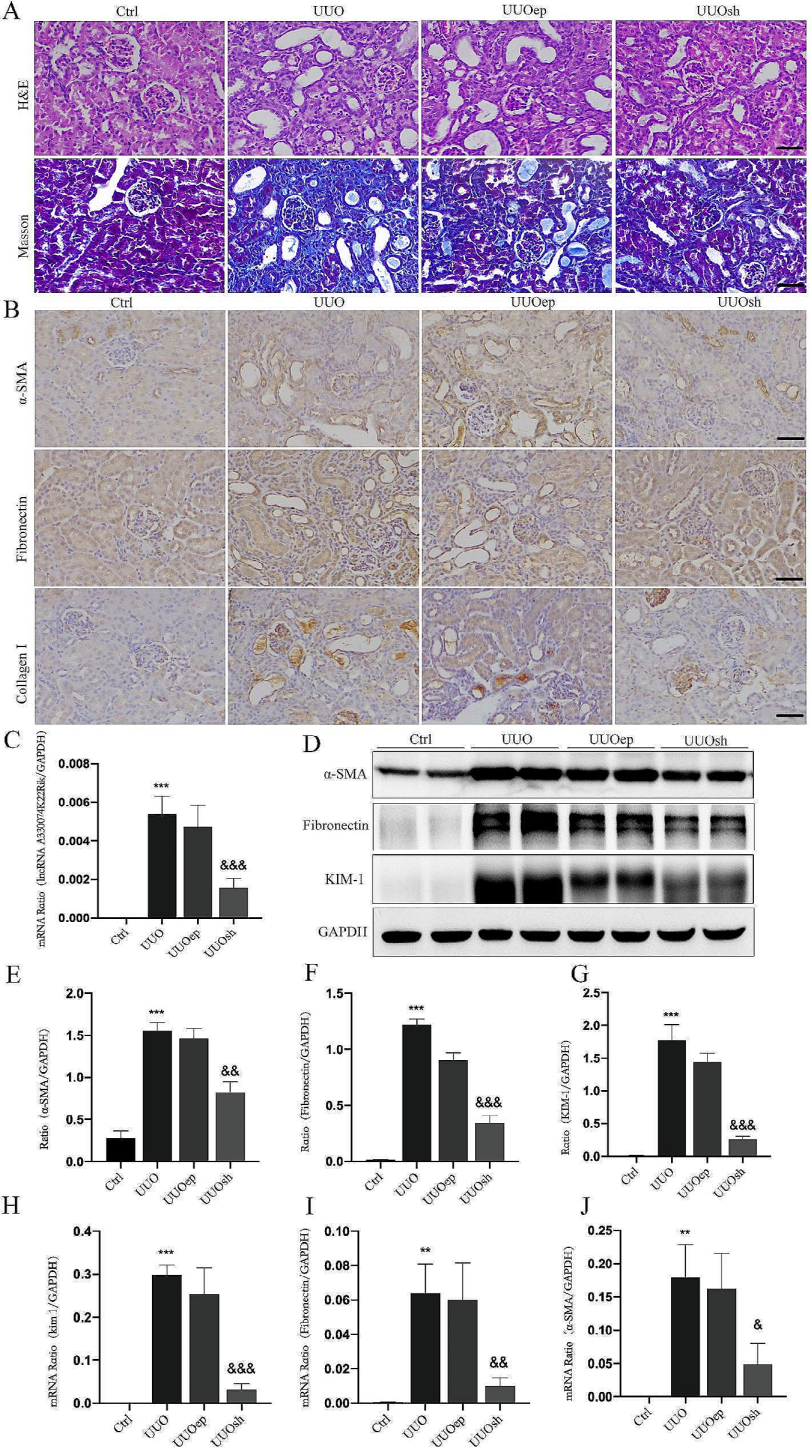
Inhibition of A33 in kidney of UUO ameliorated renal injury and fibrosis. (**A**) H&E and Masson staining in each group reveals a significant reduction in inflammatory cell infiltration and collagen fiber deposition upon LncRNA A33 knockdown. (**B**) Immunohistochemistry, WB and Real-time PCR demonstrates that inhibiting LncRNA A33 reduces fibrotic markers (α-SMA, Fibronectin, Collagen1,KIM-1). (**C**) Real-time PCR analysis of LncRNA A33 expression levels in each group confirms successful knockdown. (**H**-**J**) The mRNA expression levels were decreased in each group. (α-SMA, Fibronectin, and KIM-1) (**D**-**G**) Western blot analysis revealed the downregulation of fibrotic protein levels and renal damage markers (α-SMA, Fibronectin, KIM-1). Compared with Ctrl group, **P < 0.01, ***P < 0.001; Compared with UUO group, &*P* < 0.05, &&<0.01, &&&*P* < 0.001

In our previous study, we found that the lncRNA A33 is highly expressed in UUO kidney tissues through whole transcriptome sequencing [19]. After the successful establishment of the UUO animal model, the expression of A33 in the kidneys was significantly increased compared to the Ctrl. Subsequently, A33 knockdown plasmids were transfected into the kidneys through in situ electroporation, and real-time quantitative PCR confirmed the successful knockdown of lncRNA A33 in UUO mice. (Fig. 3C).

To validate the impact of in situ A33 knockdown on renal tissue structure, HE staining showed that compared to the UUO group, the UUOsh group exhibited mild tubular dilation and well-preserved glomerular morphology. Masson staining results indicated a significant reduction in inflammatory cell infiltration and collagen fiber deposition in the UUOsh group (Fig. 3A). Immunohistochemistry, Western blotting, and real-time PCR consistently revealed comparable outcomes. In comparison to the UUO group, the UUOsh group exhibited a significant reversal of fibrotic indicators (α-SMA, Fibronectin, Collagen1) and a noticeable reduction in pathological damage (Fig. 3B-J). These findings suggest that inhibiting A33 in the UUO kidney can ameliorate renal injury and fibrosis.

### A&P improves TGF-β1-induced fibrotic injury in pTEC cells

**Fig. 4:**
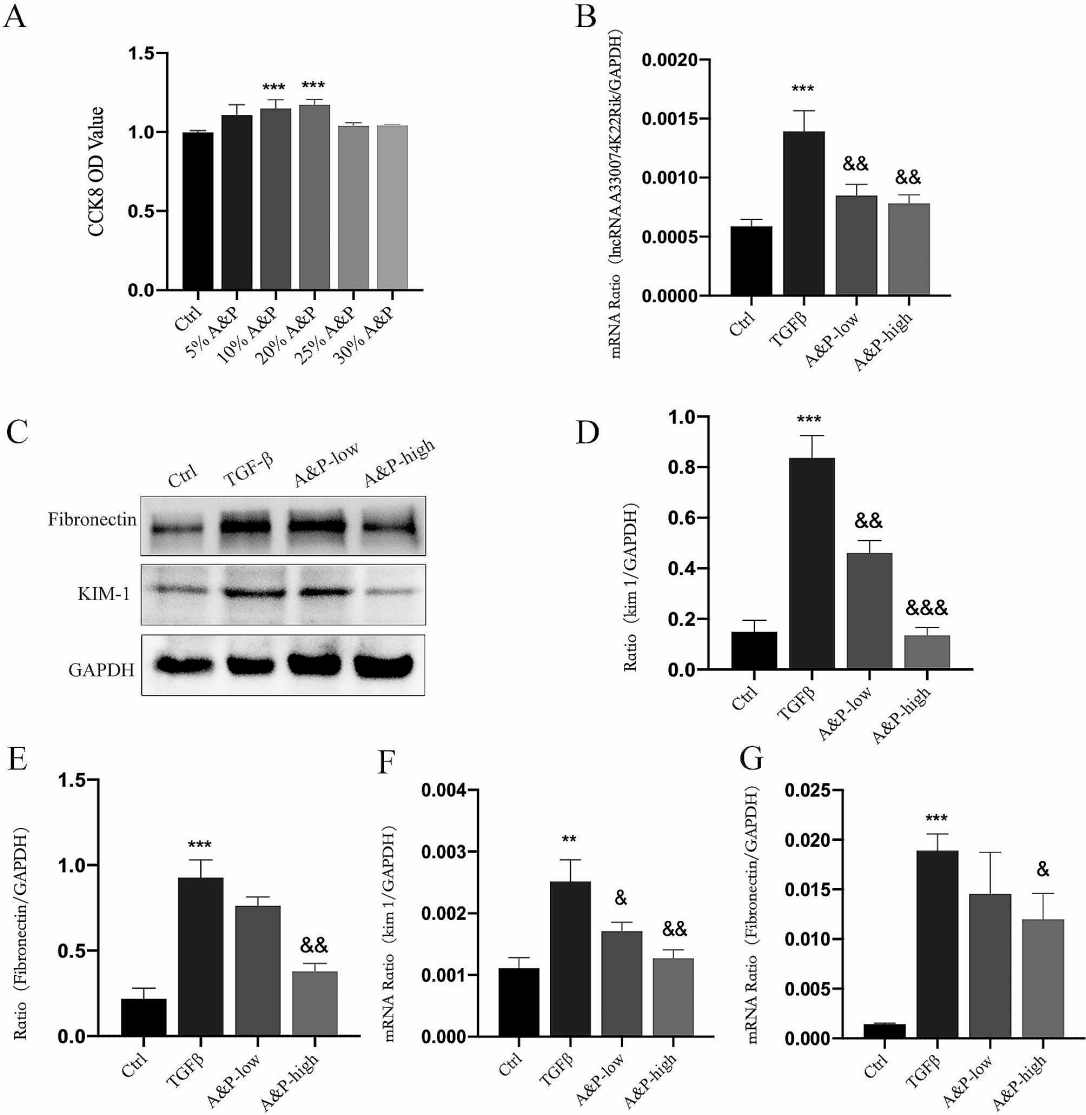
A&P Suppresses TGF-β1-Induced pTEC Cell Fibrotic Injury. (**A**) CCK-8 results illustrate the viability of pTEC cells cultured under various A&P concentrations (5%, 10%, 20%, 25%, 30%). (**B**) Based on cell viability, 10% and 20% are chosen as low and high doses, respectively. Real-time PCR analyzes the changes in LncRNA A33 mRNA levels under different concentrations of A&P treatment. (**C**-**G**) Western blot and real-time PCR analyze protein and mRNA levels of Fibronectin and KIM-1 in pTEC cells cultured with different doses of A&P. The results indicate that both low and high doses can improve cell damage, with a more pronounced effect observed at the high dose. Compared with Ctrl group, **P < 0.01, ***P < 0.001; Compared with TGF-β1 group, &*P* < 0.05, &&<0.01, &&&*P* < 0.001

Cell viability of pTEC cells treated with A&P was measured using the CCK-8 assay. As shown in Fig. 4A, when the concentration of A&P was 25%, cell viability significantly decreased, providing the optimal indication for screening the final A&P concentration gradient of 10% and 20%. Subsequently, A&P was used to treat TGF-β1-stimulated pTEC cells to validate the expression of lncRNA A33. Real-time fluorescence quantitative PCR results demonstrated a significant increase in lncRNA A33 mRNA expression in TGF-β1-induced pTEC cells, which gradually decreased with an increase in A&P concentration. Compared to the TGF-β1 group, the most significant decrease in lncRNA A33 expression was observed at a concentration of 20% A&P (Fig. 4B). To assess the impact of A&P on the expression of Fibronectin and KIM-1, consistent results were obtained in Western blotting and real-time PCR, showing a significant increase in Fibronectin and KIM-1 in TGF-β1-induced pTEC cells, which were reversed upon A&P intervention, especially at a concentration of 20% A&P (Fig. 4C-G).

### A&P inhibits iron-dependent cell death in UUO mice, relative to lncRNA A33 high expression

**Fig. 5:**
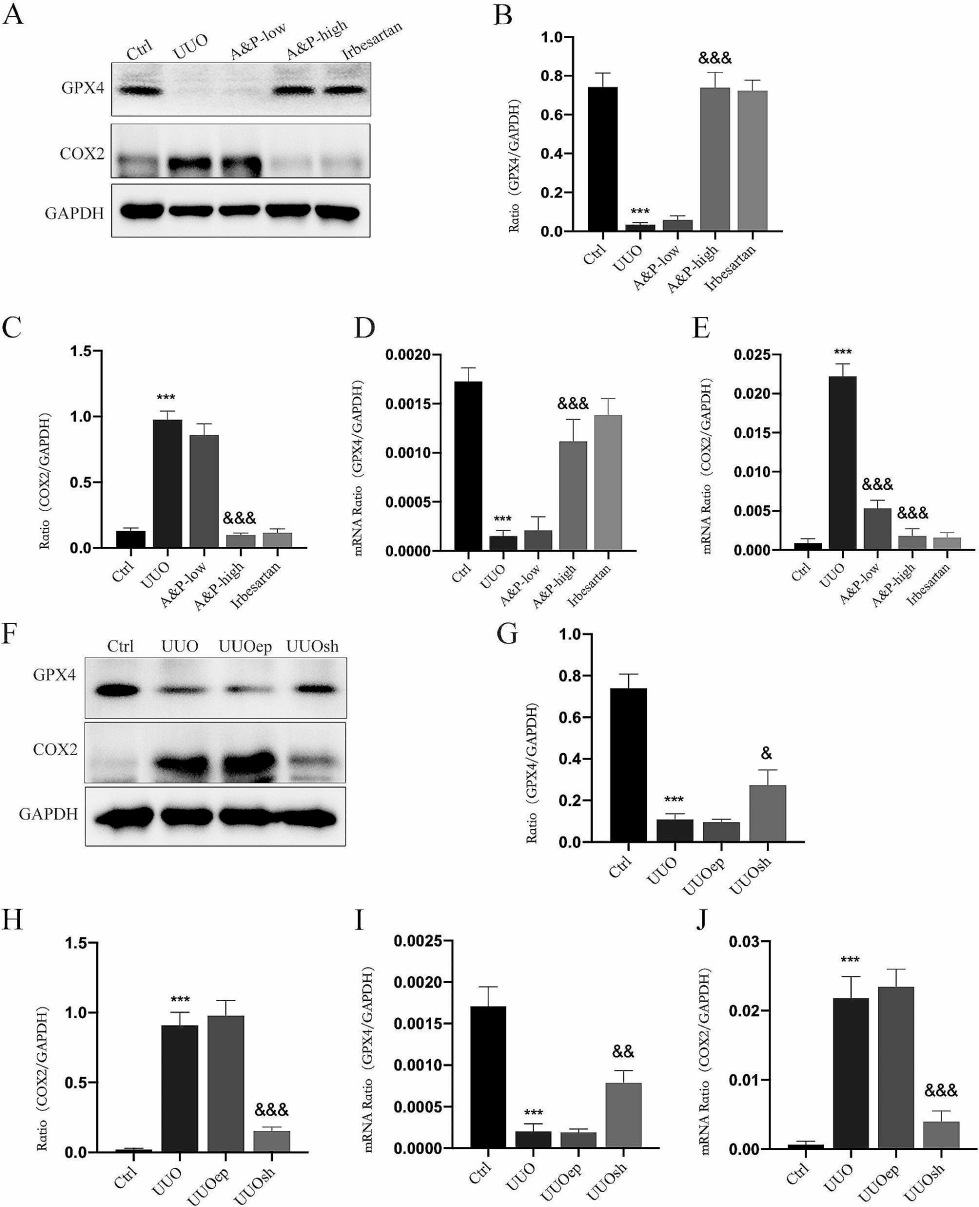
A&P Inhibits Ferroptosis in UUO Mice, and LncRNA A33 Shows Positive Correlation with Ferroptosis. (**A**-**E**) WB and real-time PCR are employed to assess changes in protein and mRNA levels of ferroptosis-related factors (GPX4 and COX2) in UUO mice. Significant reductions are observed under A&P intervention. (**F**-**J**) WB and real-time quantitative PCR examine the protein and mRNA levels of ferroptosis-related factors under LncRNA A33 knockdown conditions. The ferroptotic signals decrease with the knockdown of LncRNA A33.Compared with Ctrl group, ***P < 0.001; Compared with UUO group, &&<0.01, &&&*P* < 0.001

To further investigate the protective effects of A&P on the kidneys, we examined the expression of iron-dependent cell death factors (GPX4 and COX2) in renal tissues affected by A&P. We utilized Western blotting and real-time PCR to assess changes in the levels of proteins and mRNA of GPX4 and COX2. Specifically, GPX4 was reduced in UUO mice and reversed with A&P intervention, whereas COX2 levels were elevated in UUO mice and significantly reversed with A&P intervention (Fig. 5A-E).

Subsequently, we attempted to verify the correlation between iron-induced cell death and lncRNA A33 by examining the changes in iron-dependent cell death factor proteins under lncRNA A33 knockdown conditions using WB. Interestingly, following lncRNA A33 downregulation, the UUOsh group demonstrated a significant reversal in GPX4 and COX2 compared to the UUO group (Fig. 5F-J).

These results indicate that A&P significantly improves cellular iron-dependent cell death. Furthermore, inhibiting lncRNA A33 leads to a significant downregulation of iron-induced cell death signaling, which may represent important targets and mechanisms through which A&P inhibits UUO.

### Fibrosis and iron-induced cell death mediated by lncRNA A33 in TGF-β1-stimulated pTECs

**Fig. 6:**
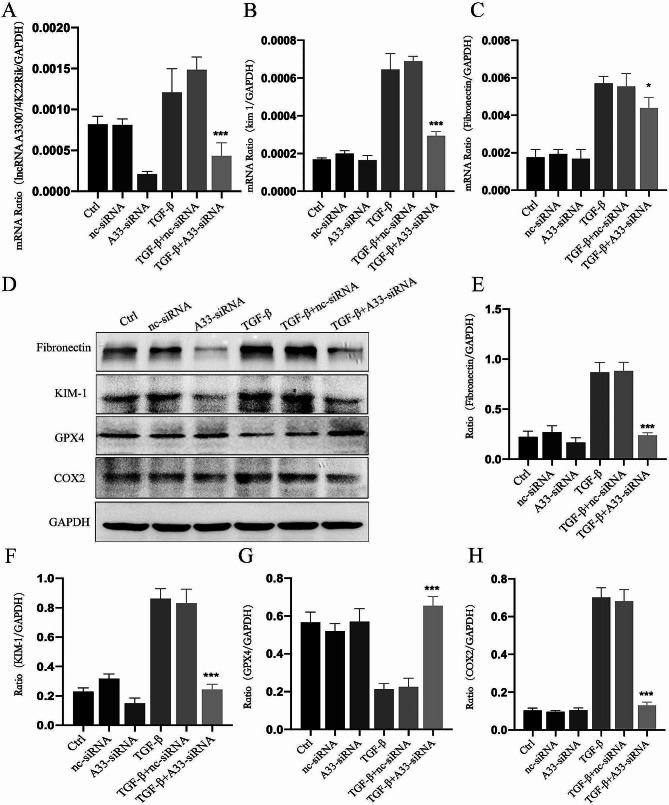
Modulation of LncRNA A33 Mediated Fibrosis and Ferroptosis Expression in TGF-β1-Stimulated pTEC. (**A**-**C**) Real-time PCR validates the downregulation of LncRNA A33 and renal injury factors (Fibronectin, KIM-1) after knockdown. (**D**) Western blot confirms protein levels associated with fibrosis and ferroptosis. (**E**-**H**) Results in pTEC cells indicate that the knockdown of LncRNA A33 reverses TGF-β1-induced fibrosis and ferroptosis. Compared with TGF-β1 group, *P < 0.05, **P < 0.01, ***P < 0.001

To investigate the role of A33 in fibrosis and ferroptosis in pTEC cells, siRNA was utilized to knock down A33 in these cells. Real-time PCR results demonstrated a significant inhibition of TGF-β1-induced A33 expression in pTECs following siRNA transfection. In comparison to the Ctrl group, there was a marked increase in A33 expression in the kidneys of the TGF-β + nc-siRNA group, whereas the TGF-β + A33-siRNA group exhibited a considerable reduction in renal A33 expression (Fig. 6A). This reduction correlated with a notable attenuation of kidney fibrotic injury (Fibronectin, KIM-1) (Fig. 6B-C). Subsequently, Western Blot results validated the expression of kidney fibrotic injury (Fibronectin, KIM-1) in the TGF-β + A33-siRNA group. Interestingly, the expression of ferroptosis markers (GPX4 and COX2) significantly improved compared to the TGF-β + nc-siRNA group. Furthermore, akin to the findings in UUO mice where LncRNA A33 was knocked down (as depicted in Fig. 5) (Fig. 6D-H). This suggests a potential attenuation of LncRNA A33’s regulation of the ferroptosis signal, indicating that ferroptosis might constitute a downstream signaling pathway of LncRNA A33. These outcomes highlight the pivotal role of inhibiting LncRNA A33 in ameliorating fibrosis and ferroptosis responses in pTEC cells. Moreover, this improvement may potentially occur through the modulation of downstream ferroptosis signals.

### A&P strongly inhibits fibrosis and iron-induced cell death *in vitro via* lncRNA A33

**Fig. 7:**
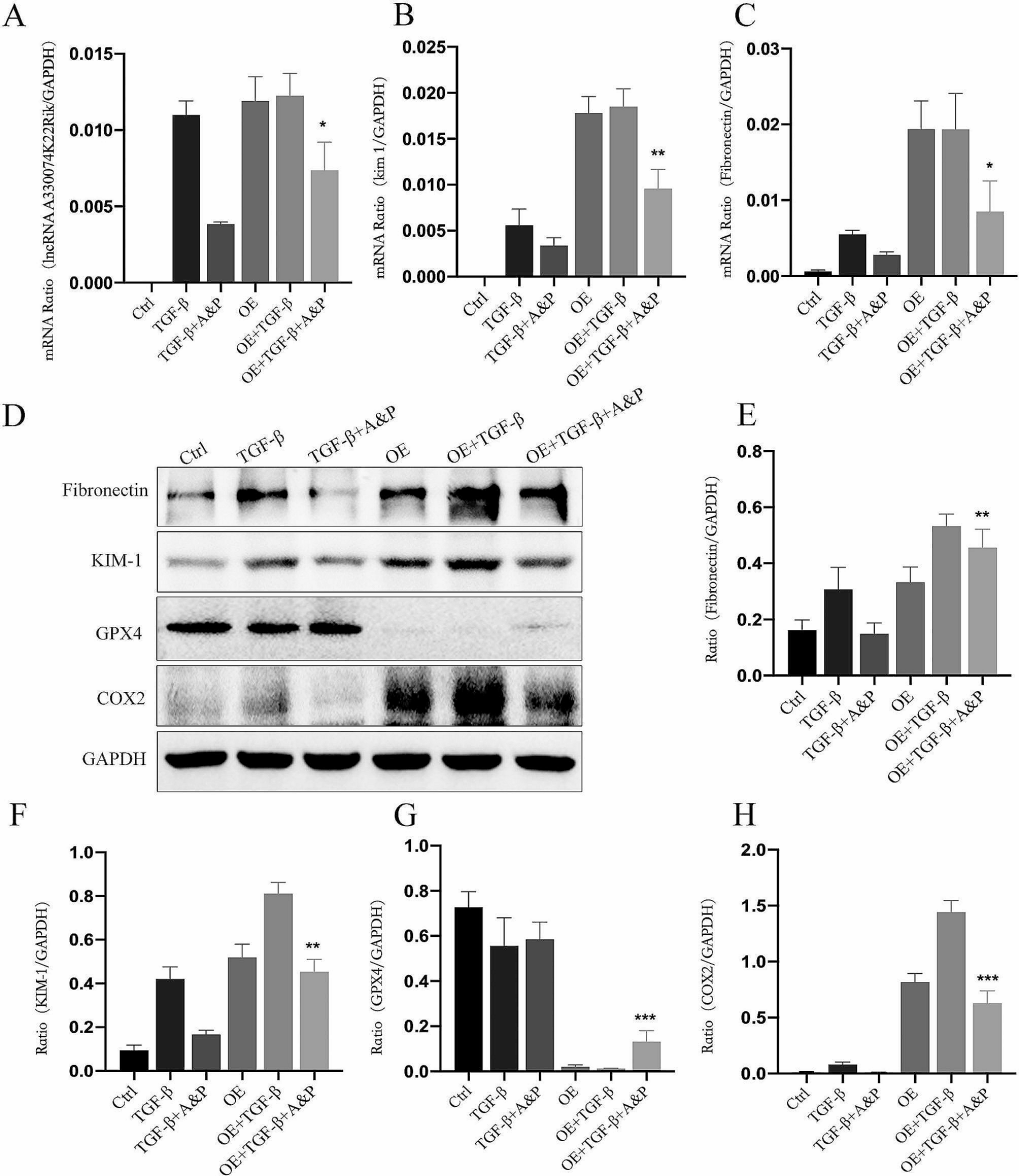
A&P Exhibits Inhibitory Effects on Fibrosis and Ferroptosis Expression Both In Vitro and In Vivo, with LncRNA A33 Potentially Acting as an Upstream Regulator of Ferroptosis. (**A**-**D**) Real-time PCR and WB results confirm a significant reduction in mRNA and protein levels associated with fibrotic injury (Fibronectin, KIM-1) in the A&P treatment group. (**E**-**H**) WB confirms that in vitro overexpression exhibits a synergistic relationship between fibrosis and ferroptosis expression with the expression of LncRNA A33. A&P demonstrates significant inhibitory effects on fibrosis and ferroptosis in pTEC cells overexpressing LncRNA A33 stimulated by TGF-β1.Compared with TGF-β1 group, *P < 0.05, **P < 0.01, ***P < 0.001

The activation of the lncRNA A33 signaling pathway is a significant mechanism involved in fibrosis, while iron-induced cell death may be a critical gene within the A33 signaling pathway. Therefore, lncRNA A33 overexpression plasmids were utilized to upregulate lncRNA A33 expression in pTEC cells, and the impact on fibrosis-related damage factors and iron-induced cell death was observed. Real-time PCR results demonstrated that plasmid transfection significantly increased lncRNA A33 expression in pTEC cells. In the TGF-β1 + A&P group, lncRNA A33 was significantly inhibited, accompanied by a reversion in the expression of Fibronectin and KIM-1. However, this inhibitory effect was attenuated after lncRNA A33 overexpression (Fig. 7A-C). Subsequently, Western blot analysis was performed to assess the changes in fibrosis-related factor protein levels. It was found that A&P notably downregulated Fibronectin and KIM-1 induced by TGF-β1 in pTEC cells and improved iron-induced cell death (GPX4 and COX2), nevertheless weakened after over-expression of lncRNA A33 in TGF-β1 induced pTEC cells (Fig. 7D-H). These findings suggest that A&P may alleviate fibrosis by suppressing the downstream ferroptosis pathway, possibly through the inhibition of LncRNA A33.

## Discussion

CKD has become a global health concern that has a hidden onset and is associated with complications such as cardiovascular accidents, leading to high morbidity and mortality rates [20]. However, effective therapeutic strategies for CKD remain limited, and current approaches, such as dialysis and kidney transplantation, fall short of preventing all complications associated with CKD [21] Consequently, a comprehensive understanding of the mechanisms underlying CKD and the development of targeted therapies is crucial. Recent investigations have unveiled the intricate interplay between ferroptosis and kidney diseases, with a particular emphasis on its pivotal role in the pathophysiology of chronic kidney disease (CKD) [22]. This study aims to explore the impact of A&P on ferroptosis signaling, elucidating the role and mechanisms of A&P in the treatment of UUO. In this study, we established a mouse model through UUO to simulate kidney fibrosis in vivo, and we used TGF-β1 stimulation of pTEC cells to establish an in vitro model. These two methods are currently considered classical approaches for establishing in vivo and in vitro fibrosis models, respectively [23]. In our previous study, we have established the detectability of ferroptosis signals in the UUO mouse model [24], and likewise in cellular models [25]. Hence, it is feasible to employ these methods in one research study.

Existing literature suggests that artemisinin (ARTs) exerts anti-tumor effects by modulating iron metabolism, generating reactive oxygen species (ROS), and activating endoplasmic reticulum stress (ERS) to regulate ferroptosis [26]. Baicalin exerts anti-oxidative stress activity through a novel Nrf2/xCT/GPX4-dependent ferroptosis regulatory axis [27]. During fibrosis cascades, ferroptosis occurs in myofibroblasts during extracellular matrix deposition and targeted regulation of ferroptosis has been shown to effectively alleviate chronic organ damage and tissue fibrosis [28]. It has been demonstrated that various traditional Chinese medicines and their active compounds can modulate ferroptosis in organ parenchymal cells, thereby exerting anti-fibrotic effects and showing promising prospects for further research.

A&P, an herbal formulation of Astragalus membranaceus and Panax notoginseng, has been developed by our research group. It has been clinically used for over a decade and has demonstrated precise efficacy, high safety, and notable therapeutic advantages. Preliminary studies have confirmed the pharmacological activities of A&P’s active components, including calycosin-7-O-glucoside, ferulic acid, and ginsenosides, and standardized the manufacturing process of the formulation [29]. The extraction process of A&P has been optimized using a comprehensive multi-index scoring method combined with an orthogonal design [30]. A&P has shown various therapeutic effects in experimental models. It not only improves structural disruptions in the colonic mechanical barrier and reduces the expression of inflammatory factors, thus preventing and treating chronic kidney disease in rats [31], but also significantly improves kidney function and pathological changes induced by cisplatin-induced acute kidney injury (AKI) in mice, inhibiting inflammation, and secretory activities in renal tissue [32]. However, the specific mechanisms of A&P’s effects on CKD are not yet clear.

Previous extensive research has revealed the significant regulatory role of lncRNAs in CKD [33, 34]. For instance, LncRNA ANRIL recruits EZH2 to the BDNF promoter region, mediating BDNF transcriptional repression, and subsequently regulating protein expression associated with endothelial function and mitochondrial dynamics [35]. Livin promotes EMT through the regulation of lncRNA-ATB [36]. LncRNA PVT1 inhibits the progression of renal fibrosis by inactivating the TGF-β signaling pathway [37]. Through high-throughput RNA sequencing of mouse renal tissues in a UUO model, a newly identified lncRNA A33 was discovered. However, there is currently no research reporting the role of lncRNA A33 in preventing or treating renal interstitial fibrosis. In this study, real-time PCR confirmed the high expression of LncRNA A33 in the UUO model. Histological examination revealed severe kidney injury, interstitial fibrosis, and partial tubular dilation in UUO mice, which correlated with the increased expression of A lncRNA A33 following UUO modeling, consistent with our sequencing results. Interestingly, in situ, electrotransfection-mediated targeted knockdown of lncRNA A33 significantly reduced its expression, as confirmed by real-time PCR, Masson staining, and immunohistochemistry. This reduction effectively alleviated renal inflammation in mice. Similarly, treatment with A&P also yielded positive results in UUO mice. Therefore, we propose that lncRNA A33 is involved in the occurrence and progression of CKD, and A&P can suppress lncRNA A33 expression in UUO, thereby exerting a therapeutic effect.

Ferroptosis is an important target gene (gene 15) in the LncRNA signaling pathway. Several differentially expressed lncRNAs (lncRNA31) have been identified in kidney fibrosis and CKD patients. In a study of A&P-regulated UUO mice, significant alterations in ferroptosis signaling were observed. Compared to the UUO group mice, mice treated with A&P showed a significant decrease in the expression of ferroptosis, which is consistent with the results obtained by in vivo targeted electroporation-mediated lncRNA A33 knockdown and in vitro siRNA-mediated lncRNA A33 downregulation. However, the opposite effect occurred when A33 was overexpressed. Interestingly, following lncRNA A33 overexpression, there was a significant increase in ferroptosis expression, and the therapeutic effect of A&P was partially counteracted by the significant elevation of fibrosis (α-SMA, Fibronectin) levels and changes in ferroptosis (GPX4 and COX2) signaling. This suggests that lncRNA A33-mediated regulation promotes cellular ferroptosis. Based on these findings, we propose that A&P may exert its therapeutic effects in CKD by modulating the activity of the LncRNA-A33 pathway to inhibit cellular ferroptosis.

Our study provides valuable insights; however, it is important to acknowledge certain limitations. Specifically, in our in vivo animal experiments, we employed electroporation techniques to selectively knock down the expression of LncRNA A33 in the kidneys, without implementing systemic gene silencing. However, overexpression of LncRNA A33 in vivo was not carried out due to external constraints. These limitations may impact the generalizability and introduce potential biases to our findings. In the context of animal experiments involving gene knockdown through electroporation, this technique demands highly specialized laboratory settings and expertise. Challenges related to efficiency and stability are inherent in electroporation technologies.

In summary, the lncRNA A33 has been identified in UUO kidney tissues and plays a crucial role in the onset and progression of CKD, exerting a promoting effect. Furthermore, A&P exhibits a protective effect against UUO and TGF-β1-induced renal tubular epithelial cell fibrotic injury. Mechanistically, A&P significantly inhibits the cell ferroptosis pathway by suppressing the expression of lncRNA A33, leading to a significant downregulation of both in vivo and in vitro fibrosis markers. In conclusion, this study demonstrates that A&P improves CKD by downregulating the LncRNA A33 to inhibit the cell ferroptosis pathway.

### Electronic supplementary material

Below is the link to the electronic supplementary material.

Supplementary Material 1

## Data Availability

The datasets generated and/or analysed during the current study are available in the corresponding author’s repository, Li Wang, at the following email address: wangli120@swmu.edu.cn.

## Abbreviations

CKD: Chronic kidney disease
ESRD: End-stage renal disease
A&P: Aastragalus Pseudo-ginseng combination
UUO: Unilateral ureteral obstruction
pTEC: Tubular epithelial cells
LncRNA A33: Long Non-coding RNA A330074K22Rik
EMT: Epithelial-mesenchymal transition
CCK-8: Cell Counting Kit-8
Ctrl: Control group
ARTs: Artemisinin
ROS: Reactive oxygen species
ERS: Endoplasmic reticulum stress
