# Supplementary figures and images for: Astragalus mongholicus bunge and panax notoginseng formula (A&P) improves renal fibrosis in UUO mice via inhibiting the long non-coding RNA A330074K22Rik and downregulating ferroptosis signaling
Source: BMC Complement Med Ther. 2024 Jul 19;24:273. doi: 10.1186/s12906-024-04557-4 (PMC11264518; doi:10.1186/s12906-024-04557-4)

Fig2

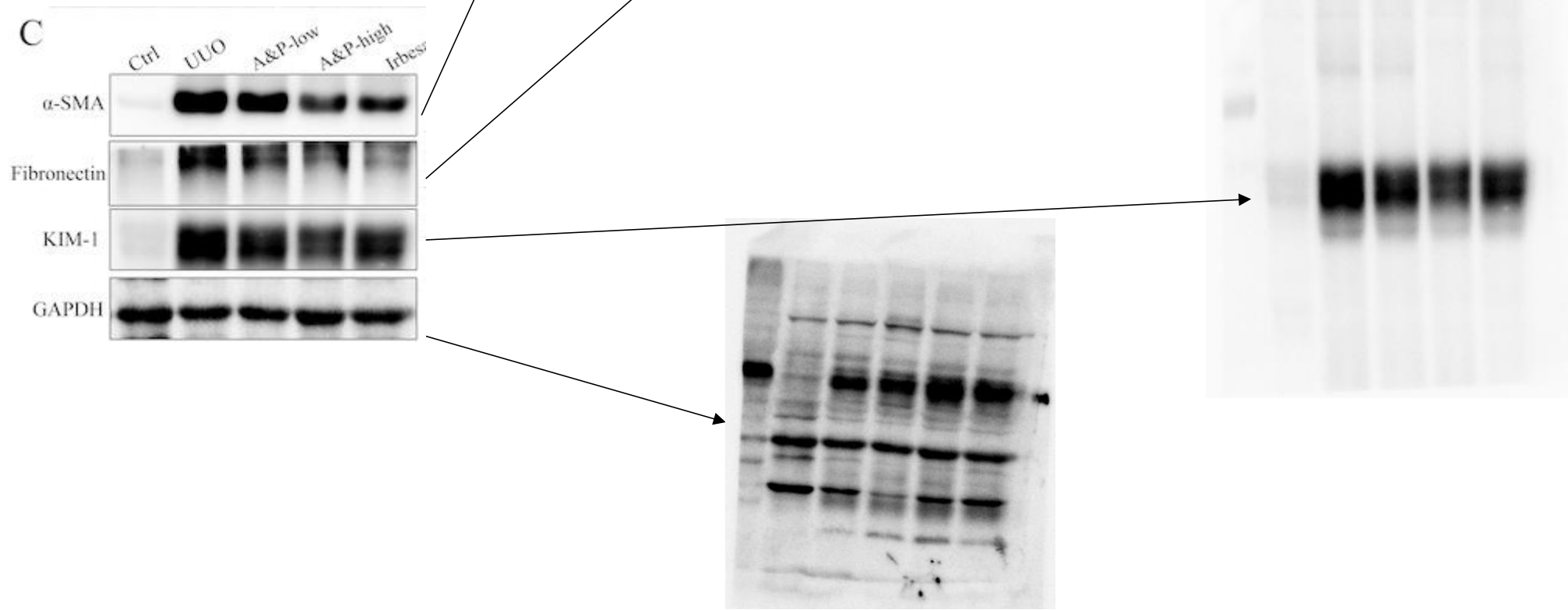

Fig3

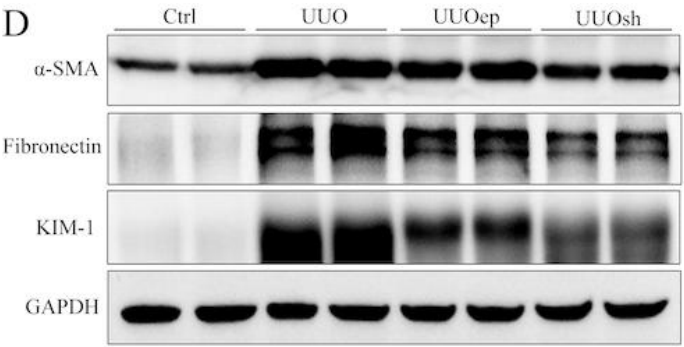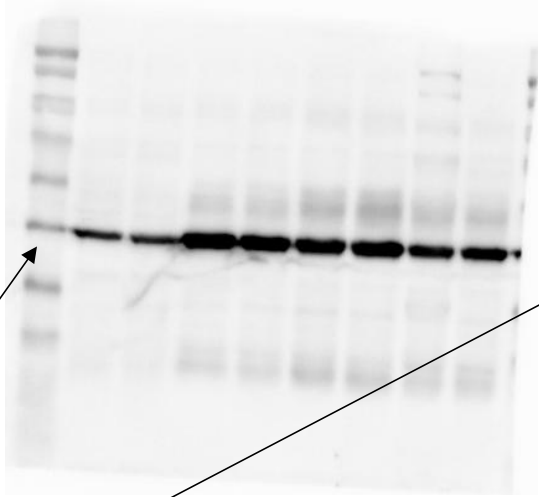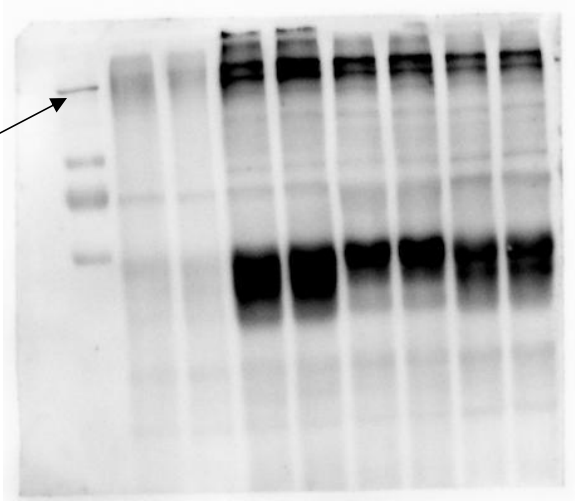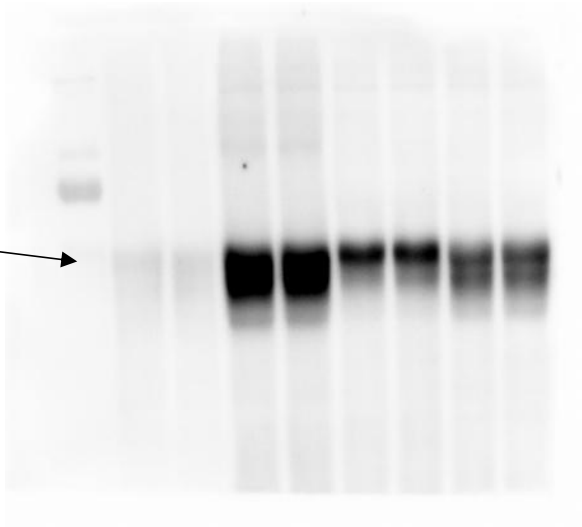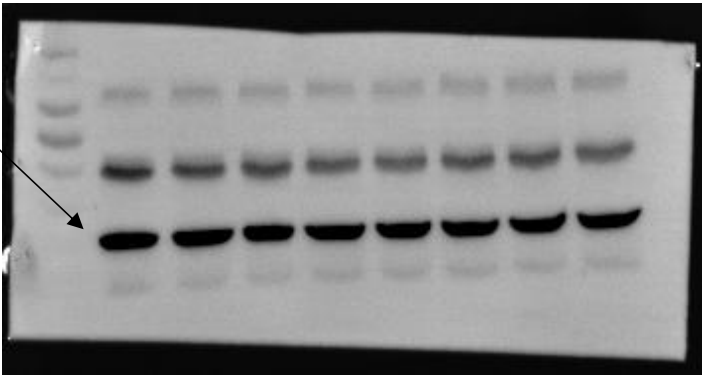

Fig4

C

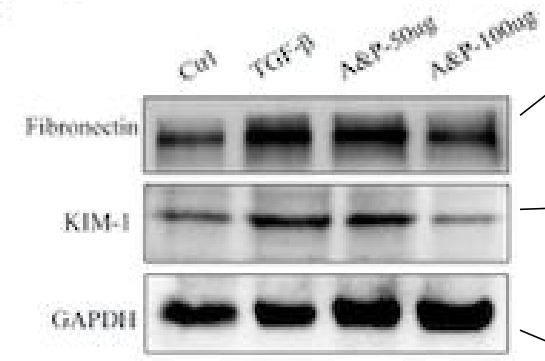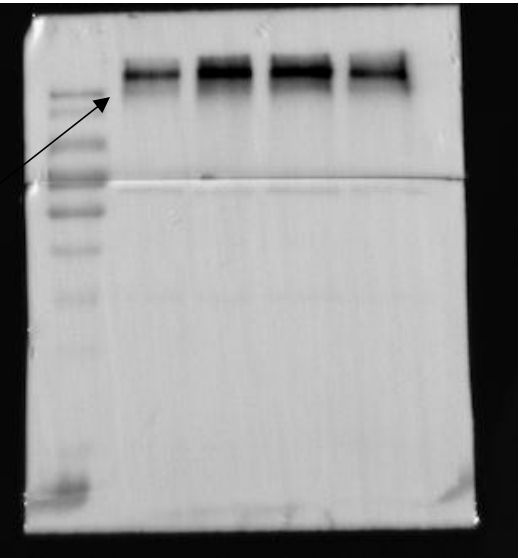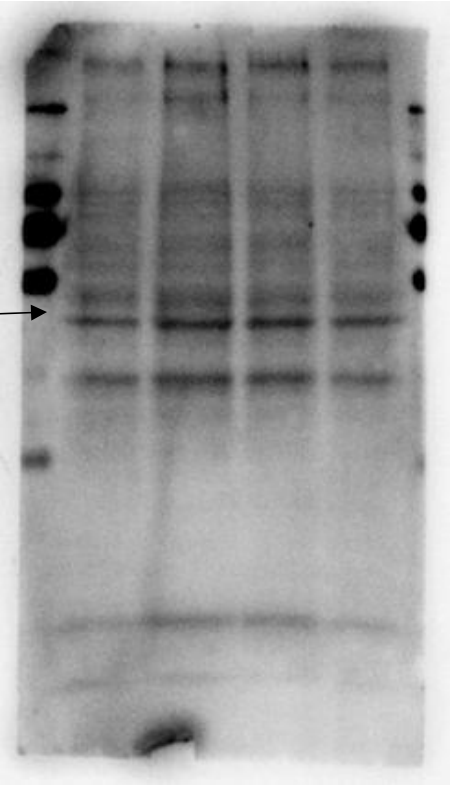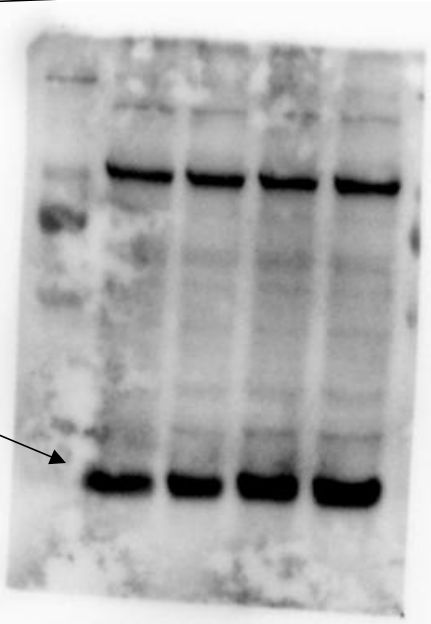

Fig5

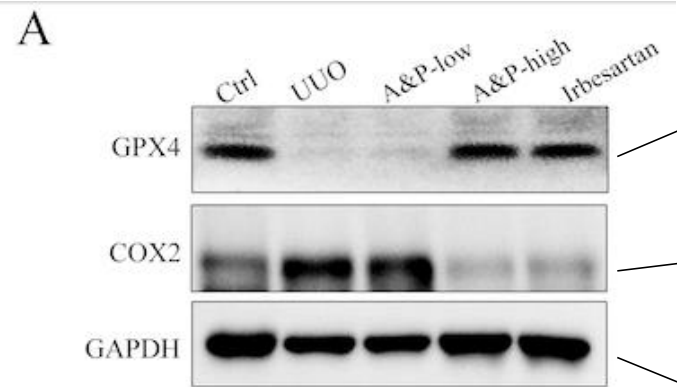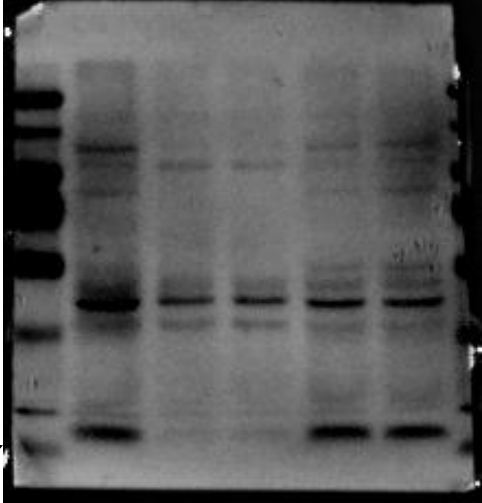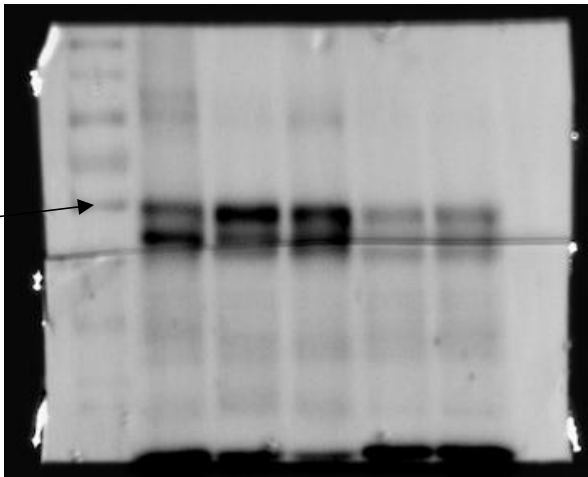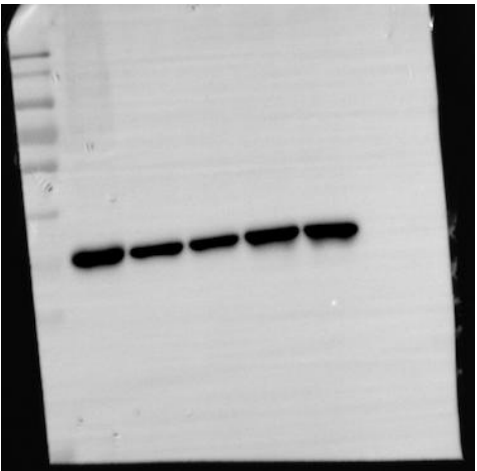

Fig5

E

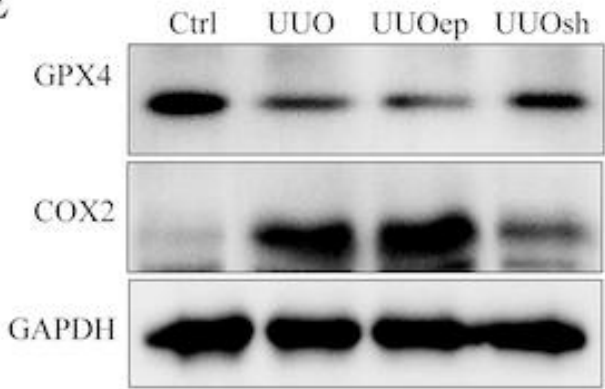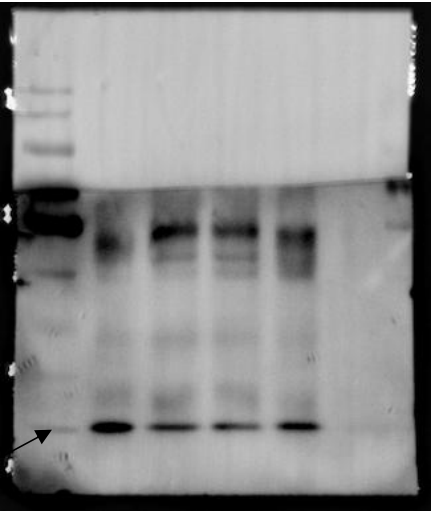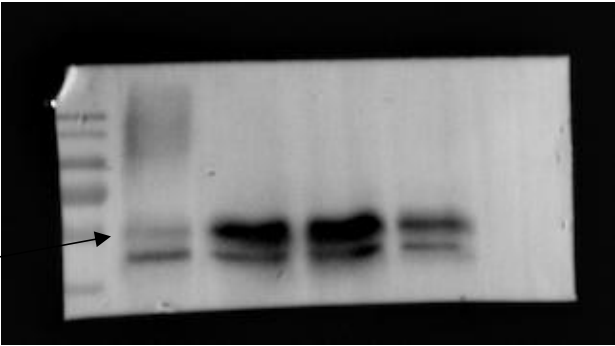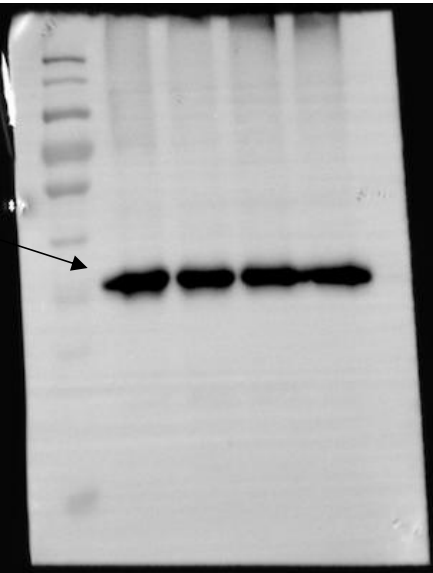

Fig6

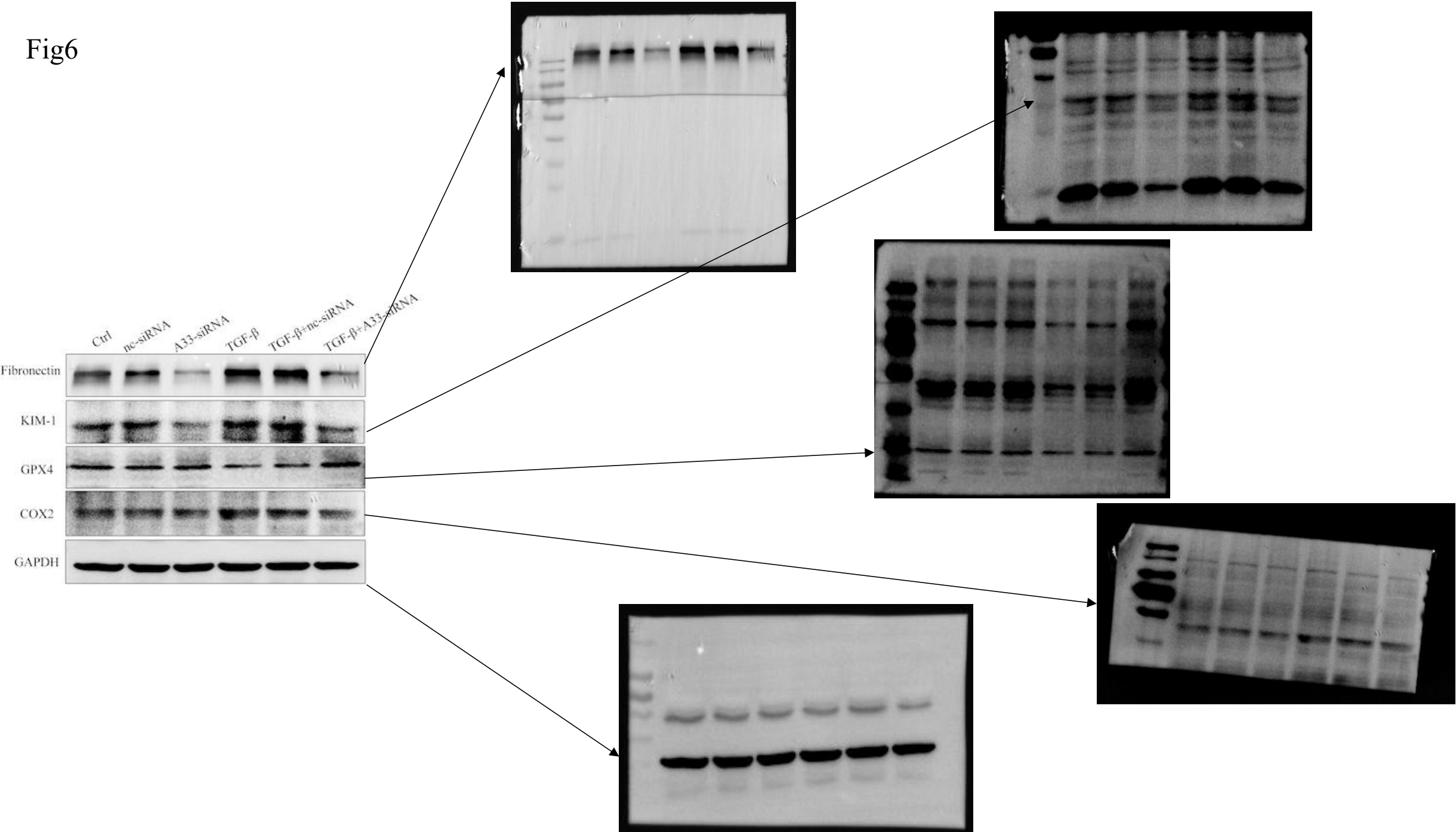

Fig7

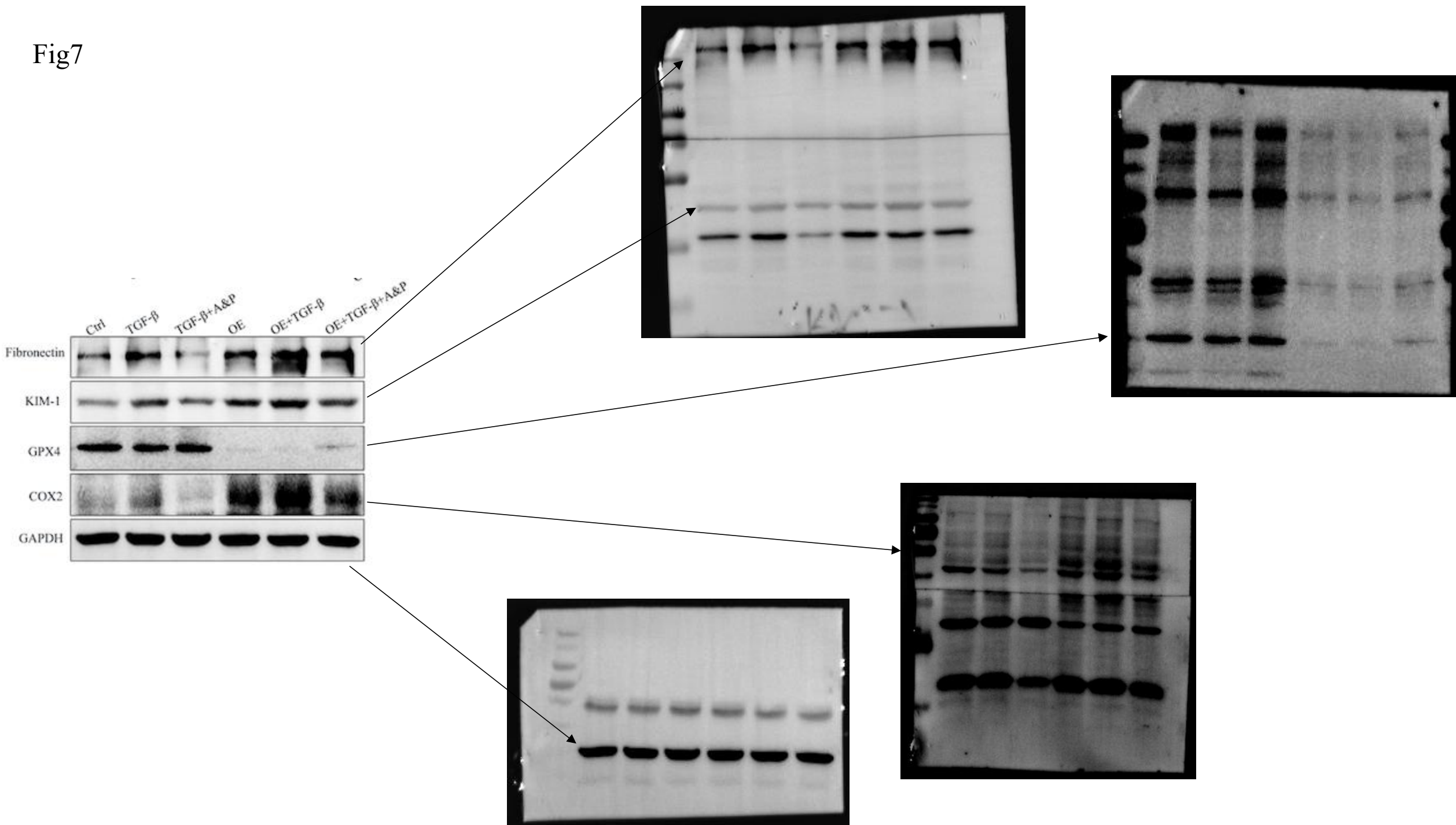

Supplement: Supplementary file 1 — Supplementary Material 1 [file 12906_2024_4557_MOESM1_ESM.pdf]
